# Figure–ground organization and the emergence of proto-objects in the visual cortex

**DOI:** 10.3389/fpsyg.2015.01695

**Published:** 2015-11-03

**Authors:** Rüdiger von der Heydt

**Affiliations:** The Zanvyl Krieger Mind/Brain Institute, Johns Hopkins University, BaltimoreMD, USA

**Keywords:** visual cortex, neural mechanism, single-cell recording, perceptual organization, object files, object permanence, attention, contour grouping

## Abstract

A long history of studies of perception has shown that the visual system organizes the incoming information early on, interpreting the 2D image in terms of a 3D world and producing a structure that provides perceptual continuity and enables object-based attention. Recordings from monkey visual cortex show that many neurons, especially in area V2, are selective for border ownership. These neurons are edge selective and have ordinary classical receptive fields (CRF), but in addition their responses are modulated (enhanced or suppressed) depending on the location of a ‘figure’ relative to the edge in their receptive field. Each neuron has a fixed preference for location on one side or the other. This selectivity is derived from the image context far beyond the CRF. This paper reviews evidence indicating that border ownership selectivity reflects the formation of early object representations (‘proto-objects’). The evidence includes experiments showing (1) reversal of border ownership signals with change of perceived object structure, (2) border ownership specific enhancement of responses in object-based selective attention, (3) persistence of border ownership signals in accordance with continuity of object perception, and (4) remapping of border ownership signals across saccades and object movements. Findings 1 and 2 can be explained by hypothetical grouping circuits that sum contour feature signals in search of objectness, and, via recurrent projections, enhance the corresponding low-level feature signals. Findings 3 and 4 might be explained by assuming that the activity of grouping circuits persists and can be remapped. Grouping, persistence, and remapping are fundamental operations of vision. Finding these operations manifest in low-level visual areas challenges traditional views of visual processing. New computational models need to be developed for a comprehensive understanding of the function of the visual cortex.

Poggio’s (1995) neurophysiological recording system, which he passed on to me when he retired in 1993, allowed the experimenter to generate visual stimuli defined either by luminance/color contrast or by disparity (‘cyclopean’ figures, more precisely, dynamic random dot stereograms). A cyclopean square, for example, would stereoscopically appear as a textured shape floating in front of an equally textured background. One day I recorded an orientation selective cell that responded to the edge of a cyclopean square (which had no luminance or color contrast) as well as to the contour of a luminance-defined square (which had no depth), and in both cases it responded selectively to the left-hand side of the figure, but not the opposite side (I could place any part of the square in the receptive field by changing its position relative to the monkey’s fixation point). For a contrast-defined figure this would be a trivial observation because the opposite sides have opposite contrast polarity, and simple cells are known to be selective for contrast polarity. But this cell had the same side preference for the cyclopean figure whose edges did not have contrast, but just stereoscopic depth. With the cyclopean figure it seemed to be selective for the direction of the step in depth, and with a luminance-defined figure for the contrast polarity of the edge. That the preferred edge conditions were on same side for both types of figure might have been a coincidence. When I encountered another cell that again preferred the same side for cyclopean and contrast squares, I flipped the colors of figure and ground for the latter, and, to my surprise, the cell still preferred the same side (although the edge contrast was now reversed). Thus, it was not contrast polarity that determined the responses. The preferred stereoscopic stimulus was the edge of a surface extending to the right of its receptive field. Was it possible that the cell responded to contrast edges only when they were part of a figure to the right of its receptive field? But how could it know that there was a figure? Through the small window of its receptive field it could only ‘see’ a contrast border.

## Border Ownership

**FIGURE 1:**
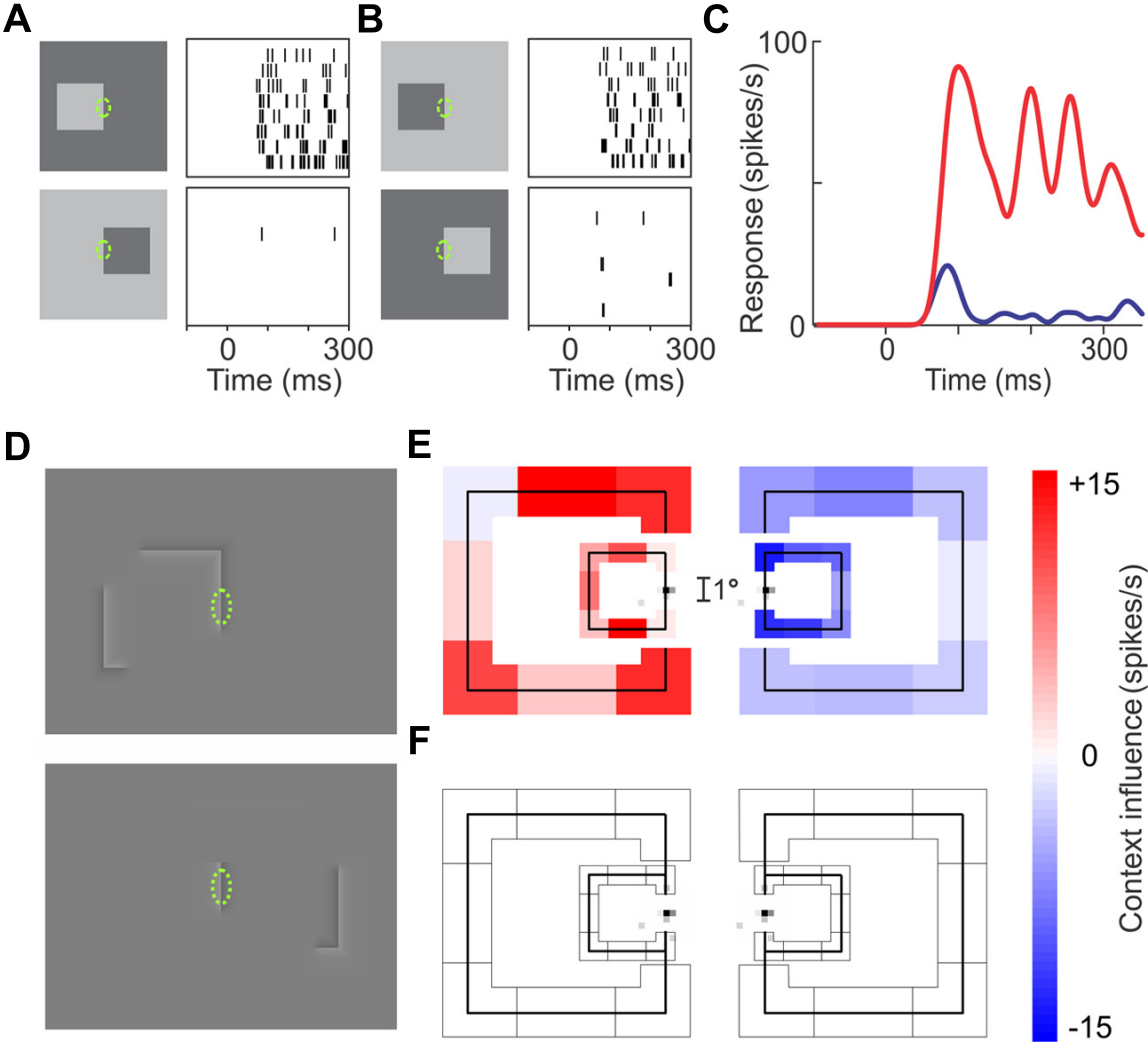
**Border ownership selectivity.** **(A–C)** Responses of an example V2 neuron. Rows of tics in **(A,B)** represent repeated responses to the stimuli shown on the left. **(A)** The responses to the right-hand edge of a bright square (top) are much stronger than the responses to the left-hand edge of a dark square (bottom) despite stimuli being identical in the receptive field (green ellipse, not part of display): the neuron is sensitive to the image context. **(B)** Same as **(A)**, but contrast reversed. Again, the cell responds more strongly when the square is to the left of the receptive field: the neuron prefers ‘left’ border ownership. **(C)** Average time course of the neuron’s firing rate for left (red) and right (blue) border ownership. Note divergence of curves right after response onset (68 ms after stimulus onset in this neuron). **(D–F)** Demonstration of the range of context integration. A V2 Neuron was examined with edges as in **(A)**, but with contour-defined squares where luminance variations were confined to a narrow seam at the contours **(D)**. The contours were broken up into eight fragments (four edges and four corners); one edge was placed over the receptive field, while the seven ‘contextual’ fragments were presented randomly in all possible combinations. **(E)** The influence of each of the contextual fragments on the responses, as determined by regression analysis. Colored shading represents the regression coefficients, red indicating enhancement, blue, suppression. Results are shown separately for left and right locations of square, and for two sizes of square (3° and 8° visual angle). The small gray specks on the test edge show the map of the neuron’s classical receptive field determined with flashing bars. The small size of this near-foveal receptive field contrasts with the large range of context integration: nearly all contour fragments to the left of the receptive field enhanced the responses, whereas contour fragments to the right suppressed them. **(F)** The result of presenting the same contextual fragments as in **(E)**, but without the edge in the receptive field: the contextual fragments alone produced no response. (Modified from Zhang and von der Heydt, 2010.)

**Figures 1A–C** illustrates this side-of-figure selectivity in a cell recorded in the secondary visual area V2. The cell responds strongly to edges of figures located on the left of its receptive field (**Figures 1A,B**, top), but hardly at all to edges of figures on the right (bottom), and it does so for light-dark edges (**Figure 1A**) as well as dark-light edges (**Figure 1B**). Note that the edges in the receptive field are identical between the vertically aligned displays, but the responses differ dramatically. Apparently, despite the small size of its receptive field, the cell knows the edge belongs to a larger object and that the object is located on the left.

**FIGURE 2:**
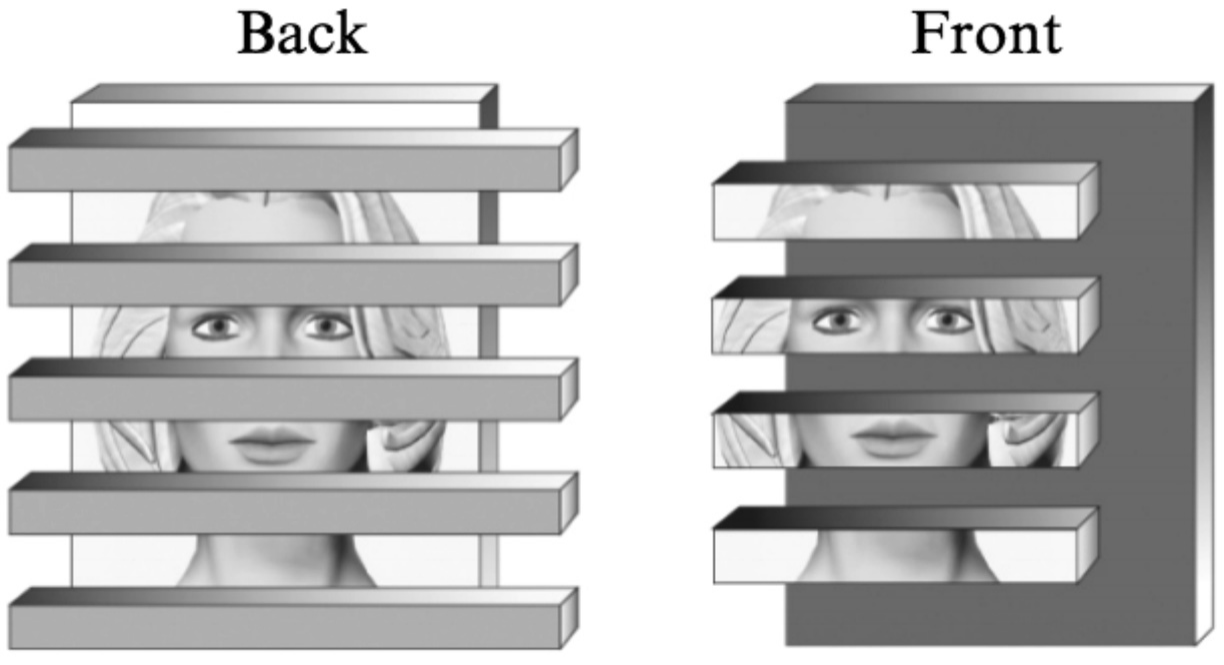
**Illustration of the face recognition experiment of Nakayama et al. (1989).** When the face is in back, it is easy to identify, but when the face strips are in front, the system assigns their borders to the strips, thus grouping the face information with the horizontal borders, and face identification becomes difficult. The illustration uses pictorial tools to depict depth, in the experimental study depth was defined by stereoscopic presentation. (Reproduced with permission from Nakayama and Shimojo, 2009.)

We have termed this ‘border ownership coding’ (Zhou et al., 2000). Indeed, I was primed by the perceptual studies of Nakayama et al. (1989, 1995), who showed that the role of a contour in perception depends on how it relates to the adjacent regions: the ‘intrinsic’ contours of a object contribute to its perception, whereas ‘extrinsic’ contours produced by other, occluding objects are excluded. For example, a face that is partially covered by horizontal strips can be recognized easily when the face is stereoscopically behind the occluding strips (**Figure 2**, left). However, recognition is difficult when the visible parts of the face are stereoscopically in front (right). In this case, the borders between strip and face regions are grouped with the face regions, making them appear as separate objects. In contrast, when the occluding strips are in front, they own those borders, allowing the face fragments to regroup behind the strips. Thus, the system assigns borders to the side that is nearer, and it seems to do so before the information is passed on to the object recognition stage.

From the comparison of contrast-defined and disparity-defined figures, as described above, it became clear that the selectivity for side-of-figure relates to the process of interpreting a 2-dimensional image in terms of a 3-dimensional world. The disparities define unambiguously what is in front and what is in back, and the occluding contours belong to what’s in front. The contrast-defined figures are ambiguous; the figure region could be an object or could be a window through which a background surface can be seen. Recordings from area V2 showed a clear correlation. The ‘near’ side of the preferred stereoscopic edge was generally the same as the preferred side for the contrast-defined figure without disparity cue (Qiu and von der Heydt, 2005). In other words, despite the lack of specific depth cues, the cortex interprets the contrast-defined figure as a foreground object, rather than a window or a region of different reflectance on a flat surface.

To do this, the cells rely on the global distribution of contours in the vicinity of the classical receptive field (CRF). A closed contour is of course strong evidence for the foreground object interpretation. But even fragments of a closed contour can suggest the presence of an object. Experiments show that, while a contrast edge in the CRF is necessary to elicit a response, the response is modulated by the presence of additional edges which are compatible with an object on one side or the other. The role of such contextual edges was probed by presenting fragments of a putative object contour (**Figures 1D–F**). The contours of squares were broken up into eight fragments (four edges and four corners), one edge fragment was placed on the CRF (green dashed ellipse), and the influence of the contextual fragments on the response was determined. In **Figure 1E**, the effect of each fragment is indicated by the color of shading (results for two sizes of squares are illustrated). One can see that most fragments on the left (preferred) side enhanced the responses, whereas fragments on the right side suppressed them. The plot at the bottom (**Figure 1F**) shows the result of presenting the contextual fragments without an edge in the CRF: there was no response. Thus, the contextual elements do not drive the cell, their influence is purely modulatory. The results of this experiment reveal the large range of the context influence compared to the tiny CRF.

One might think that neural border ownership selectivity is merely about the coding of borders, adding a directional tag to each contour segment. But further experiments showed that the underlying process goes much deeper, and that studying border ownership coding provides insight into the way the system defines what is an object.

## Object Structure

**FIGURE 3:**
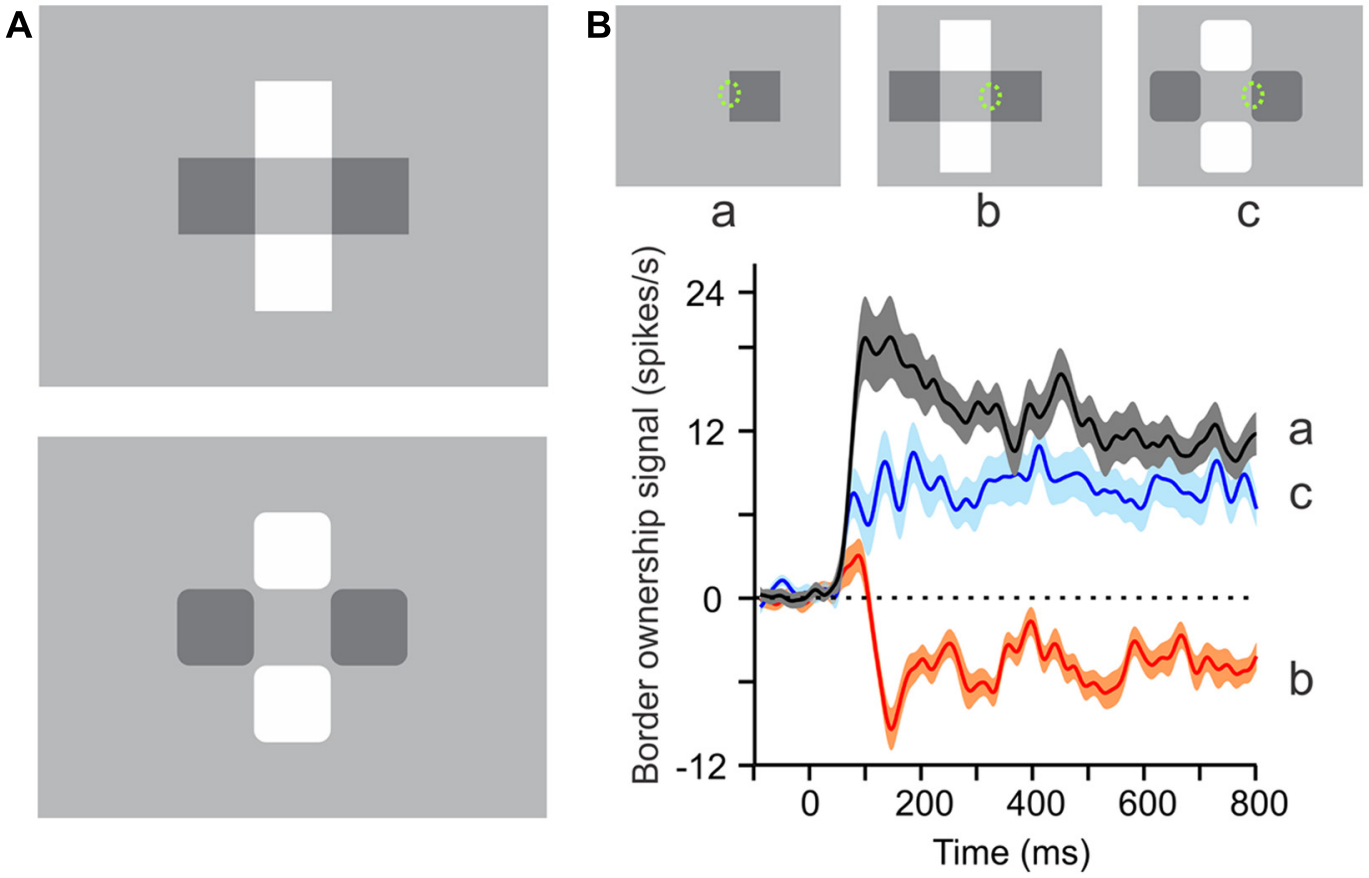
**Border ownership signals correlate with perceptual organization.** (**A**, Top) This configuration of two light and two dark squares is generally perceived as a pair of crossed bars in transparent overlay, or as a light bar with a shadow on it, or a dark bar crossed by a beam of light. However, when the corners of the squares are rounded off, only the individual squares are perceived (**A**, bottom). **(B)** Curves show the average border ownership signals (difference between preferred and non-preferred side responses) for the three conditions shown at the top, where a *green ellipse* marks the location and approximate size of receptive fields (not part of display). Note the reversals of perceived border ownership of the marked edge between *a* (owned right), *b* (owned left), and *c* (owned right). The neural border ownership signals reverse sign accordingly. (Modified from Qiu and von der Heydt, 2007.)

The following experiment (Qiu and von der Heydt, 2007), exploits the phenomenon of perceptual transparency (**Figure 3**). While an isolated square perceptually owns its contour on all four sides, one can take away the ownership on one side by adding three squares to create a configuration as in **Figure 3A** (top). This configuration is generally perceived as two crossed bars in transparent overlay. Note that perception here assigns the interior edges to the bars. Thus, adding the squares has flipped border ownership on one side of the original square. But when the corners of the squares are rounded off (**Figure 3A**, bottom), the four squares are perceived as individual objects and ownership of the interior edges returns to the squares. The displays at the top of **Figure 3B** show how ownership of the border marked by a dotted ellipse is flipped by the image context. In the experiment, this border was placed in the receptive field of the recorded neuron.

The neural signals faithfully reflect these perceptual reorganizations. For the single square, the border ownership signal for the edge in the receptive field is positive (black curve in **Figure 3B**), corresponding to ‘right’ border ownership, but for the transparent configuration, the signal turns negative (red curve), indicating ‘left’ border ownership. For the squares with rounded corners, the signal is again positive (blue curve). Thus, the border ownership signals reflect the way the brain interprets the visual stimulus in terms of objects. Most of the neurons in V1 and V2 represent local border segments, and the imposed border ownership modulation shows how the cortex groups these segments to contours of objects. This early contour assignment determines the shape processing at subsequent stages (Bushnell et al., 2011).

## Object-Based Attention

Why would the brain need to group features at this early stage? One reason, we argue, is to provide a structure for selective attention. Recordings from the visual cortex have shown that attention tends to enhance responses in neurons representing the attended stimulus compared to distracter stimuli. A popular explanation is the spatial attention model which assumes that attention enhances the responses at a single behaviorally relevant location in the visual field (‘spotlight of attention’). However, in natural images, which are generally cluttered because 3D scenes are projected onto a 2D receptor surface, a simple spotlight of attention is of little use. The system is generally interested in objects, not image regions, and to select information in an object-based manner a structured representation is needed.

Recordings made while the monkey was performing a selective attention task indicate that the same mechanisms that produce border ownership modulation provide the structure for object-based attention (Qiu et al., 2007). Two findings are important:

First, border ownership assignment occurs with or without attention. When three objects are displayed simultaneously, one of which is designated the target for a shape discrimination task, border ownership modulation occurs at all three objects and is only slightly stronger at the attended object compared to the ignored objects (Qiu et al., 2007).

**FIGURE 4:**
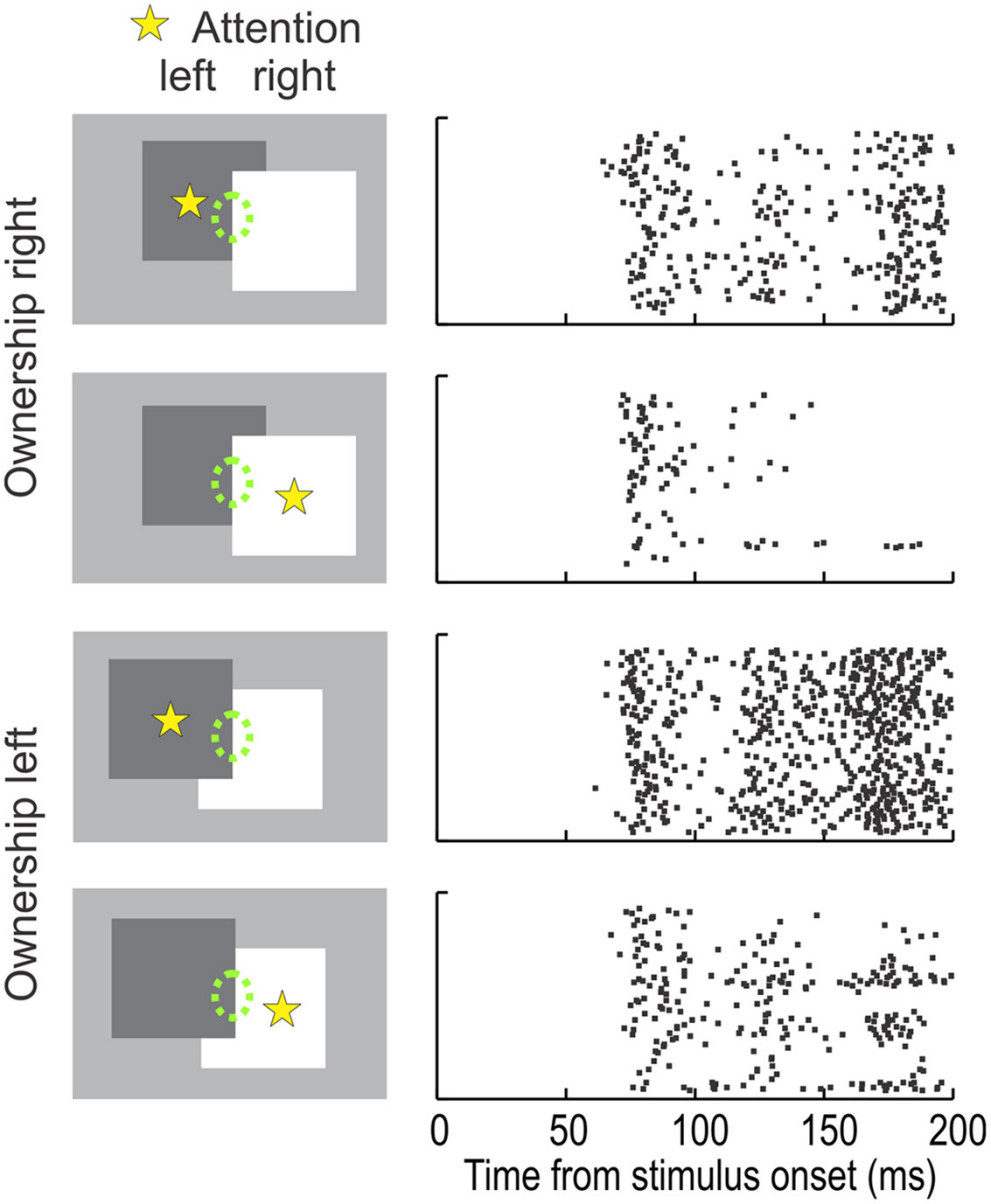
**The influence of attention on the responses of a typical border ownership selective neuron.** The receptive field of the neuron (*green ellipse*, not part of display) was placed on the border between two overlapping figures. In one configuration (*rows 1 and 2*) this ‘occluding edge’ is owned by the square on the right, in the other configuration (*rows 3 and 4*) it is owned by the square on the left. Attention was controlled by having the monkey perform a shape discrimination task with one of the figures (according to preceding instruction). The attended figure is marked here by a *yellow asterisk* (not part of display). ‘Left’ border ownership produced stronger responses than ‘right’ border ownership in both attention conditions. Interestingly, the attention effect was also asymmetric. Attention on the left figure enhanced the responses compared to attention on the right figure, irrespective of border ownership (compare rows *1* and *2* and rows *3* and *4*). This asymmetry of the attention influence was systematic across the population: attention enhanced responses on the preferred border ownership side, relative to attention on the non-preferred side. (Modified from Qiu et al., 2007.)

Second, many border ownership neurons in V2 are modulated also by attention, and there is an interesting link. In the individual neuron, the attentional modulation depends not only on the receptive field being in the focus of attention, but also on the location of the attended object relative to the receptive field. This can be seen clearly when looking at the representation of the border between two overlapping figures (**Figure 4**, green dashed ellipses indicate receptive field). The direction of occlusion determines border ownership. In the top two displays, the right-hand figure owns the border, in the bottom two displays, the left-hand figure. For each border ownership condition, either the left or the right figure could be the target of attention (yellow asterisk). The raster plots on the right illustrate the responses of an example neuron. Comparing the responses for the same location of attention (e.g., rows 1 and 3, or rows 2 and 4) one can see that the neuron preferred left border ownership. However, the strength of the responses depended also on the side of attention. Attention to the left figure produced stronger responses than attention to the right figure, irrespective of the border ownership condition. Thus, the experiment revealed an unexpected asymmetry of the attention effect. In each neuron, attention enhanced the responses on one side, but suppressed them on the other side (relative to the mean level; the experiment did not include a ‘no attention’ condition). Note also that in the neuron of **Figure 4**, the attentional enhancement was on the left side, the same as the preferred side of border ownership. In fact, this was the rule: across the population, the ‘enhancement side’ of attention tended to be the same as the preferred side of border ownership (Qiu et al., 2007).

## A Neural Grouping Model

**FIGURE 5:**
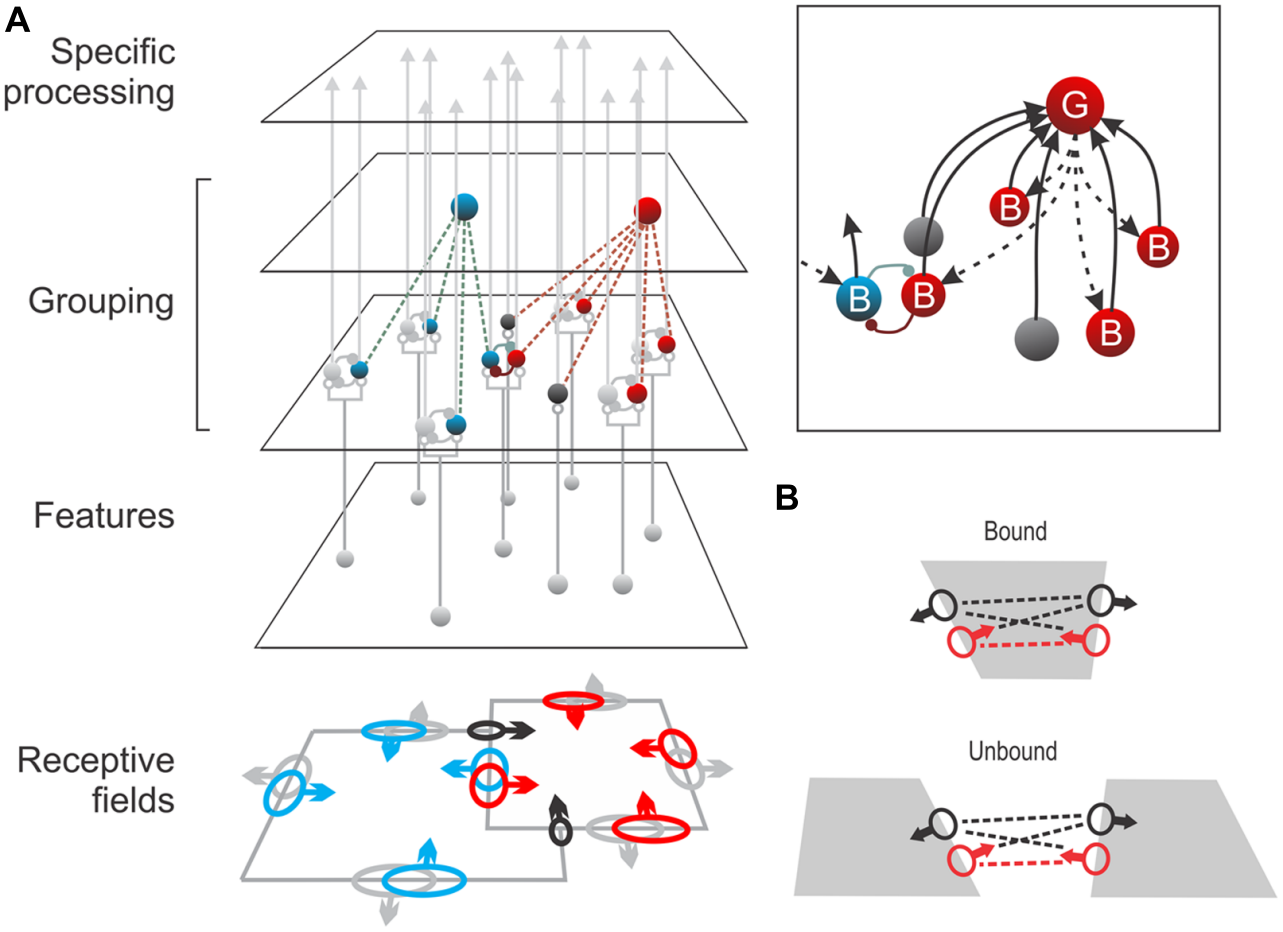
**A neural grouping model.** **(A)** Edge-selective neurons at the *Feature* level activate two populations of border ownership neurons (*B*-cells), one for each side of the edge. These neurons send signals to *specific processing* areas such as inferotemporal cortex, but they have also collateral projections to ‘*Grouping* cells’ (*G*-cells) which sum their signals, collecting edge signals in co-circular configuration. *G*-cells, by feedback, enhance the responses of the same *B*-cells (*Inset, dashed arrows*). Each *B*-cell is exclusively connected to *G*-cells on one side; thus, the enhancement makes the *B*-cell border ownership selective for that side. This is indicated by arrows on the *receptive fields* of the corresponding color. *G*-cells also sum responses of end-stopped cells (*black*) signaling local occlusion features like T-junctions which are indicators of border ownership. Assuming that top-down attention targets the *G*-cells, the model also provides a parsimonious explanation of object-based attention. **(B)** The model predicts that two *B*-cells that are connected to the same *G*-cell (*red dashed line*) will exhibit increased synchrony of firing when both are stimulated by the same object (*bound*) compared to activation by different objects (*unbound*), whereas *B*-cells that do not share common *G*-cells (*black dashed lines*) will show no increased synchrony. A recent study confirmed exactly these predictions (Martin and von der Heydt, 2015). (Modified from Craft et al., 2007.)

At this point it will help to consider a simple circuit model (**Figure 5**). This model was conceived to reconcile the wide range of context integration with the short latency of border ownership signals (**Figure 1**). This is a problem for realistic modeling because, in primates, V1 and V2 are very large areas (in fact the largest in cerebral cortex, Adams et al., 2007), and both are retinotopically organized. Therefore, in the typical border ownership test, the context information that indicates where the figure is located is only represented far from the recorded neuron (Sugihara et al., 2011). Given the slow conduction of intracortical fibers, propagating the context information within V1 or V2 cortex would lead to conduction delays on the order of 100 ms, in contradiction to the experimentally observed onset of border ownership signals which occurs as early as 10–35 ms after the response onset (Sugihara et al., 2011, see examples in **Figures 1C** and **3B**). The model of **Figure 5** solves this problem by postulating a feedback loop through a higher-level area. Because several higher-level areas (such as V3, MT, V4, TEO, etc.) are close by and since the connections to and from these areas consist of white-matter fibers which are 10-times faster than intracortical fibers (Girard et al., 2001), the expected conduction delays in this scheme are compatible with the experimental latencies.

The model assumes that the border ownership neurons (‘B-cells’) receive their driving input from ordinary edge selective neurons (simple or complex cells) and send their main projections to specific processing stages such as V4 and inferotemporal (IT) cortex, but also send collateral fibers to hypothetical grouping cells (‘G-cells’). The G-cells sum the input of many B-cells and, via back-projections, enhance the responses of the same B-cells (**Figure 5**, inset). The G-cells have fixed summation templates (the Craft et al., 2007, model assumes annular templates summing ‘co-circular’ edge signals). Each B-cell is exclusively connected to G-cells on one side, and when a figure is present on that side, some of these G-cells will be strongly activated and their feedback will enhance the responses of that B-cell. Thus, the feedback from G-cells makes the B-cells selective for figure location. The side of the G-cell connections determines the B-cell’s border ownership preference, as indicated by arrows on the receptive field symbols in **Figure 5.** B-cells with coincident receptive fields, but opposite side preference (pairs depicted in blue and red) are assumed to inhibit each other. In the special case of two partly overlapping figures, the contours would activate G-cells on either side about equally. However, G-cells also sum inputs from end-stopped cells (depicted in black) which respond to occlusion features like T-junctions that indicate the direction of occlusion (Heitger et al., 1992). In the situation illustrated in **Figure 5A** this would bias the activity in favor of the red G-cell. Experiments indicate that G-cells receive input also from other kinds of neurons that signal direction of occlusion, e.g., neurons selective for stereoscopic edges (von der Heydt et al., 2000; Qiu and von der Heydt, 2005) and dynamic occlusion (von der Heydt et al., 2003).

The main argument for predicting the circuit of **Figure 5** is that border ownership preference is a fixed property of the neurons. As far as we can tell, neurons do not change their side preference even after hundreds of intervening responses to a variety of stimuli. Thus, a piece of contour with a given location and orientation is represented by two groups of neurons, and, for a given side of ownership, one group is facilitated while the other is suppressed. Psychophysical and fMRI experiments showing that the two groups can be adapted independently indicate that border ownership is coded similarly in human visual cortex (von der Heydt et al., 2005; Fang et al., 2009). This opponent coding scheme differs from other proposed figure–ground models in which neurons represent the figure status of a region by their activity, e.g., by enhanced/suppressed firing, coherent oscillations or firing synchrony. In such coding schemes each neuron can represent either figure or ground.

An essential feature of the grouping model (**Figure 5**) is that it includes the basic circuitry needed for top-down selective attention. We just assume that the top-down attention signal targets the G-cell representing the object of interest, boosting its activity relative to the other G-cells. This raises the response gain in the corresponding B-cells. Thus, the system can enhance large numbers of edge signals by activating just a few G-cells and thereby select the distributed contours of an object.

With the model of **Figure 5**, it is easy to understand the peculiar asymmetry of the attentional modulation found in V2 neurons (**Figure 4**). Because each B-cell is connected unilaterally to G-cells on one side of it’s receptive field, it will be enhanced only by attention to an object on that side. Attention to an object on the other side will enhance its opponent B-cell, producing inhibition. Thus the observed correlation between side of attentional enhancement and side of border-ownership preference is a simple consequence of the connectivity in **Figure 5.**

The grouping cell model has an advantage over simple spotlight models of attention. The latter would work for foreground objects, but not for partially occluded objects. Because it simply selects all the edge signals under the spotlight, focusing on the left square in **Figure 5** would enhance all the bounding contours, including the occluding contour. The selected edges would have the shape of an L, although the object is really a square. In contrast, in the grouping model, the G-cell of the foreground figure (red) is activated strongly because it receives input from the B-cells on the contour (red) as well as from end-stopped cells responding to the T-junctions. The strong feedback from that G-cell facilitates the red B-cell on the occluding contour, which in turn inhibits the blue B-cell. Thus, when attention targets the blue G-cell, it will enhance only the blue B-cells corresponding to intrinsic contours, while the blue B-cells on the occluding contour are suppressed. Thus, attention can work in cluttered scenes. The model converts the difficult problem of object-based feature selection into a simple spatial selection at the G-cell level (Mihalas et al., 2011).

## Evidence For Grouping Cells

Searching for direct evidence for the existence of grouping cells, Anne Martin in my lab analyzed spiking synchrony between border ownership cells (Martin and von der Heydt, 2015). The grouping cell model implies that two B-cells that are connected to the same G-cell receive the same feedback spike trains. This should lead to increased synchrony of firing in the two B-cells. The prediction is highly specific (**Figure 5C**): only B-cells whose border ownership preferences are consistent with a common object (cells connected with red dashed lines) receive feedback from common G-cells, but not cells with inconsistent preferences (black dashed lines). The experiments revealed synchrony exactly as predicted: when stimulated by a common object (‘Bound’ condition), consistent pairs showed increased synchrony, but not when stimulated by two different objects (‘Unbound’ condition). In contrast, inconsistent pairs showed no synchrony in either condition. These results are strong evidence for the grouping cell model. We think that the activation of grouping circuits corresponds to the representation of ‘proto-objects’ in perception (see Discussion).

## Object Permanence

One characteristic of perceptual objects is continuity (object permanence). When an object is briefly occluded by a foreground object and then reappears, it is perceived as the same object. A token seems to persist. Persistence is also apparent in contour grouping. In Rubin’s (1921) vase-face demonstration, for example, one interpretation is typically perceived for a few seconds before it gives way to the other interpretation, and similar for the Necker cube. Object organization has inertia.

Philip O’Herron in my lab searched for persistence in border ownership signals. As the borders between vase and face regions remain assigned to one side or the other for seconds, the question was if border ownership signals would show similar persistence. In one experiment, Philip presented the edge of a square in the receptive field of a neuron, as in the test of **Figures 1A,B**, but after a brief period, the display was converted into a bipartite field, leaving an ambiguous edge in the receptive field. In some trials the initial figure presentation was on the left side, in other trials on the right side (as in **Figures 1A,B**, top and bottom), but in the end, there was the identical ambiguous edge. The border ownership modulation persisted in the ambiguous phase for about a second. If the initial figure presentation had been on the preferred side, the cell kept firing at a high rate, but if the initial presentation had been on the non-preferred side, the cell remained suppressed (O’Herron and von der Heydt, 2009).

**FIGURE 6:**
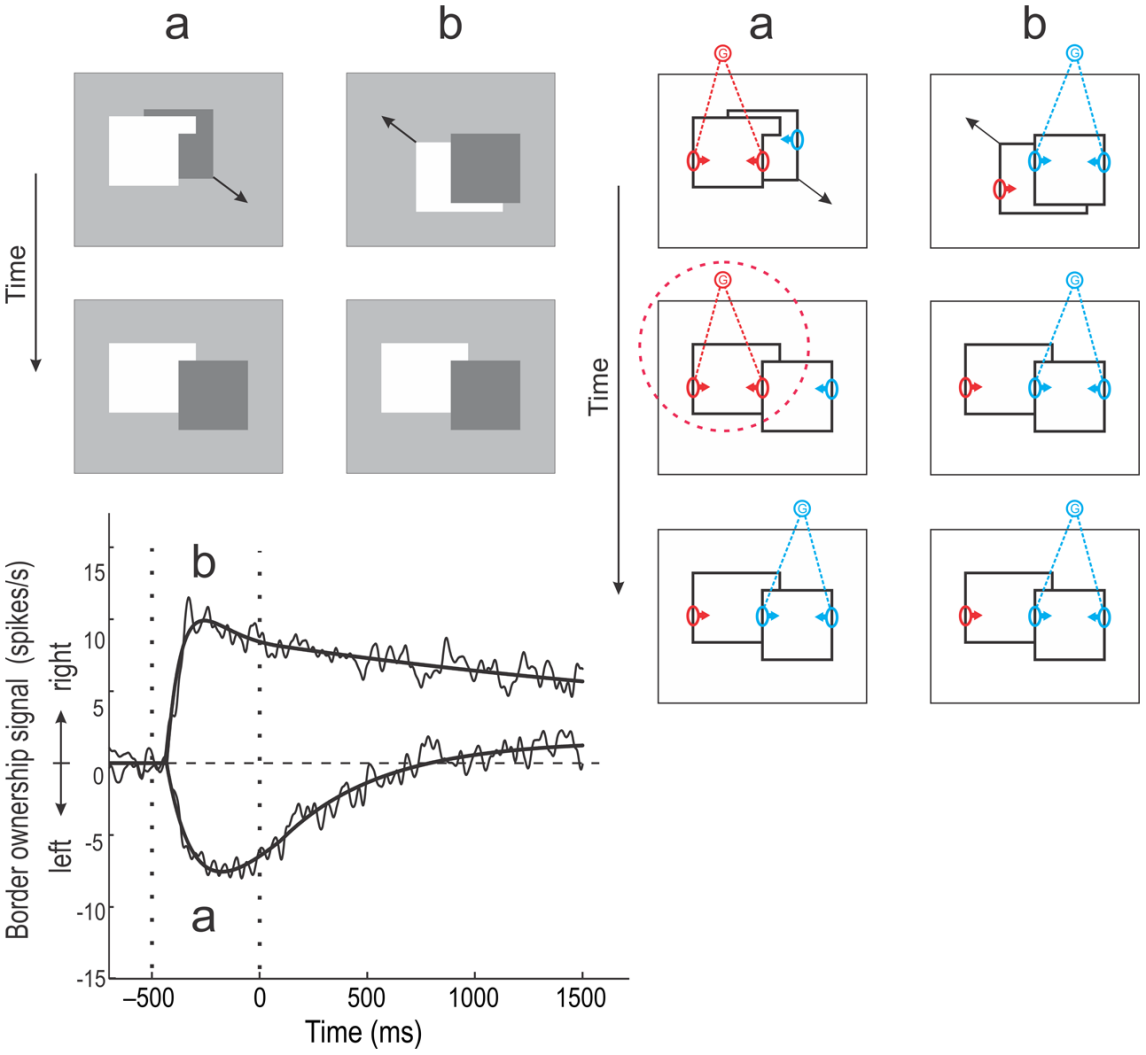
**Object continuity and persistence of grouping.** **(Top left)** Two kinds of movement displays were used. In *a*, an inverted L shape initially occluded part of a dark square (*top*). The square then moved smoothly to a new position, resulting in the configuration shown *below*, which is typically perceived as a dark square occluding a white rectangle. Thus, the perceived direction of occlusion (border ownership) changes after the termination of movement. In sequence *b*, the dark square initially occludes the white shape and the latter then moves to a new position, resulting in the same final configuration as in *a*. **(Bottom left)** Average border ownership signals (difference between preferred and non-preferred side responses) for the two display sequences. In condition *b*, the border ownership signal shoots up shortly after the figures are presented (left vertical dotted line), reaching a peak and then decaying slowly. In condition *a*, the border ownership signal first turns negative, indicating ‘left’ border ownership, and then slowly drifts toward positive values. The vertical dotted line at time 0 indicates the end of the movement. At this time, the displays in the two conditions become identical, the only difference being the history of events. However, the border ownership signals remain different for at least 1500 ms. **(Right)** The Schematic illustration of the events in terms of the grouping cell model. In condition *b*, none of the borders changes assignment. During the movement, dynamic cues (accretion/deletion) as well as static cues (T-junctions, compact shape of square) define ‘right’ border ownership at the occluding contour. After the termination of movement, the static cues continue to activate the G-cell corresponding to the square (*blue*), keeping the occluding contour assigned. In condition *a*, dynamic cues and T-junctions initially indicate the left figure to be in front, activating the *red* G-cell most strongly. But when the movement terminates, the dynamic cues disappear and the static cues now indicate ‘right’ ownership for the occluding contour. However, the experiment showed that the border ownership neurons kept signaling ‘left’ for about 800 ms, indicating that the elevated activity in the G-cell persisted (*red dotted circle*). The persistence of border assignment despite the reversal figure–ground cues indicates that the grouping mechanisms have memory (see Discussion). (Modified from O’Herron and von der Heydt, 2011.)

The following experiment demonstrates the relation to continuity in object perception (**Figure 6**), (O’Herron and von der Heydt, 2011). In each trial, two overlapping figures were presented, one of which then moved smoothly to a new position, as indicated by oblique arrows in the top displays. In (a), the foreground figure has the shape of an inverted L and the background figure, a square, is moved to fill the concavity of the L as shown below. In the final configuration, the square is generally perceived as overlapping the other figure (which seems to change from an L shape to a rectangle extending behind the square). We were interested to learn how border ownership neurons would represent the border between the two figures. At the beginning, the border is perceptually assigned to the L shape, but in the end, it is taken over by the square. How would the border ownership signals behave? For comparison, a display sequence was presented in which the border did not change ownership (b). In both (a) and (b), the final configuration was identical. In fact, everything the monkey saw was identical after the movement stopped, the only difference being the display history.

The graph below shows the border ownership signals (averaged over cells) recorded in the two conditions. In condition (b), the signal turns positive after figure onset (left vertical dotted line), indicating ‘right’ border ownership, and remains positive (gradually weakening): the border is assigned to the square. In condition (a), the signal initially goes negative, indicating that the border is assigned to the L shape. At the end of the movement (right vertical dotted line), the signal remains negative and only slowly creeps up to positive values. Note that after time 0 the displays in the two conditions were identical, but the two signals stayed separate for at least 1.5 s, the end of the monkey’s fixation period (the signals would presumably converge eventually).

The sequence of events in this experiment are illustrated in terms of the grouping cell model on the right of **Figure 6.** In condition (a) the L shape strongly activates a G-cell (red) that enhances all its edges, including the border between the two shapes, because movement and T-junctions indicate that it is a foreground object. At the end of the movement, the original T-junctions disappear and two new T junctions appear indicating the opposite relationship: the L shaped region is now likely to be background. However, the border ownership signals for the central border do not change immediately. Apparently, the ‘proto-object’ of the L shape (encircled with red dotted line) persists and continues to claim the central edge as its contour. Perhaps G-cells have intrinsic persistence and the G-cell on the left side keeps firing at a higher rate than the one on the right side despite the cues being reversed, for about 800 ms, after which the right G-cell slowly takes over. In condition (b), the right G-cell (blue) dominates throughout because compact shape and T-junctions all indicate the square to be the front object for the entire duration of the trial.

## Object Tracking And Remapping

Proto-objects would be of little use if they only lived in fixed in retinal coordinates. Once contours are grouped in one retinal location, they should stay grouped after the object moved to a new location. When a figure is flashed at one position on a display, and subsequently at a different position, we do not perceive two objects, but one that moved. The system relates the actual visual input to what has been processed the moment before. Similarly, when the eyes make a saccade, the system should be able to relate the new visual input to the object representations established before the saccade.

Since we had found that the border ownership signal for an edge persists when the edge is isolated from its context, we wondered if the isolated edge would retain its assignment after moving to a new location. Will the border ownership modulation emerge at the new location? We carried out two experiments (O’Herron and von der Heydt, 2013). In the first, a square figure was presented whose contours were outside the receptive field of the recorded neuron. Then, the display was stopped down to show only one edge of the square in a symmetrical circular window. Although we did not observe responses (the edge was not in the receptive field), we knew that a border-ownership signal had emerged and persisted in the neurons that were activated by the edge. Next, a saccade was induced (by moving the fixation point) that brought the receptive field on to the edge. The neuron started to respond after the saccade, and indeed, the strength of the ensuing response depended on how the edge had been owned in the initial display! The response was strong when it had been part of a figure on the neuron’s preferred side, but weak when it had been owned by a figure on the other side. Thus, border ownership signals ‘remap’ across saccades. Similar remapping was found in the other experiment in which the monkey kept fixating and the ambiguous edge was moved to the receptive field.

## Discussion

The above review, I hope, makes a convincing argument for early grouping mechanisms that play a fundamental role in the vision process. Border ownership selectivity, as observed in areas V1 and V2 of the visual cortex, is not just about assignment of borders; it reflects a process that allows the system to define and represent objects before they are recognized or sorted out by attention. The existence of a pre-attentive stage of organization has long been postulated by Gestalt psychologists (see Kanizsa, 1979 for an excellent explication of the Gestalt ideas). Indeed, the main tenet of Gestalt psychology is that, what we see, and what we attend to, are not the signals of photoreceptors, nor the elementary features signaled by the Hubel–Wiesel filter stage, but structures formed by an early process of organization. We call these ‘proto-objects’. They do not correspond exactly to the objects as projected into the eyes and may not be identical to the final percept that subjects report. Proto-objects reflect an early guess about the object composition of a scene. In the above experiments, neurons were tested with relatively simple geometrical shapes. However, recent studies show that the same neurons also signal border-ownership consistently in images of complex natural scenes, and again, with short latency (Williford and von der Heydt, 2014). The demonstration of grouping at the neural signal level is perhaps the strongest and most direct evidence for mechanisms of organization. While psychological and theoretical studies can infer such mechanisms, we can see them at work in the functioning visual cortex (though of non-human primates).

I have reviewed selectively studies of border ownership coding because they give a coherent and rather detailed picture of early grouping in the visual cortex, but these studies by no means stand alone. Figure–ground organization was first demonstrated in V1 by Lamme (1995), who compared the responses of neurons to a texture when it was figure with the responses to the same texture when it was ground: the responses were enhanced in the figure condition relative to the ground condition. This enhancement occurs independently of the tuning properties of the neurons (Zipser et al., 1996), and with some delay after response onset (later than the border ownership signals). Lamme and coworkers also attribute the response modulation to feedback from higher centers (Roelfsema et al., 2002), but find that attention plays a critical role (Poort et al., 2012). Using Lamme’s paradigm with motion-defined figures, Jones et al. (2015) found strong figure enhancement even at subcortical levels. Grouping in V1 is also apparent when monkeys perform a mental curve tracing task (Roelfsema et al., 1998). Upon focusing attention on one end of a curve response enhancement spreads along its entire representation. In this paradigm the effects of grouping and attention cannot be separated. However, when a monkey is cued to attend one element of an array, attentional enhancement spreads automatically to other elements according to classic Gestalt grouping rules: for example, from one bar to others that are collinear (Wannig et al., 2011). When monkeys are required to detect a line embedded in a random texture, grouping of collinear line segments produces response enhancement in V1 neurons (Li et al., 2006), and this enhancement was found to depend on feedback from area V4 (Chen et al., 2014). As shown above for border ownership signals (**Figure 6**), the Lamme-type figure enhancement also persists after the figure–ground cues are removed (Super et al., 2001). This persistence depends on endogenous attention, whereas border ownership signals seem to persist even without attention (O’Herron and von der Heydt, 2009). All these findings indicate that feature grouping occurs early in visual cortex. How exactly the results from the different paradigms relate to each other still needs to be clarified.

Why does the system need grouping at early stages? The prevailing concept of visual processing is the hierarchical model according to which object recognition proceeds in stages. Examining the ventral stream from V1 to anterior IT cortex, one finds increasing size of receptive fields, increasing shape selectivity and increasing position tolerance (Zeki, 1978; Desimone et al., 1985; Tanaka et al., 1991; Connor et al., 2007; Rust and DiCarlo, 2010). An IT neuron might respond to the presence, in a specific arrangement, of two or three contour segments each with a specific curvature (Brincat and Connor, 2004). Neurons at the highest level might be selective for even more complex combinations of features. Such neurons are highly efficient in classifying objects. Isn’t that all we need to explain object perception? I argue the answer is “No”: Classification is not representation. Classification (recognition) can be based on a few critical features which may be a small fraction of all the features that make up an object. A car can be detected based on certain features of shape, but the response of such a detector would not indicate the color of the car. By contrast, a representation of an object is a data structure that enables the system to deliberately read out any feature of an object (at least tentatively) that might be of interest. Because visual objects generally consist of many visual features, it is obviously impossible to represent objects by IT-style feature selective neurons because of the problem of combinatorial explosion.

The function of grouping cells is different from that of higher-order feature selective neurons. G-cells point to the feature neurons of an object, but do not represent the features. For example, when the contours of a red triangle activate a G-cell, top-down attention mechanisms can selectively enhance its edge signals by enhancing that G-cell and suppressing others. And because a large fraction of the edge selective cells in V1 and V2 are also color selective (Friedman et al., 2003), the selected edge signals will transmit not only shape, but also color information to subsequent stages. The G-cell need not be color selective, and its back-projections may target edge neurons of all colors indiscriminately, but the red triangle activates only a subset of color selective edge neurons, and only those are enhanced by the feedback (because the feedback is purely modulatory, it does not activate neurons that are not driven by the actual stimulus, **Figure 1F**). Thus, a non-color-coded G-cell can select signals that enable subsequent stages to compute the color of the triangle.

With simple annular fuzzy G-cell templates the model sketched in **Figure 5** effectively zooms in on objects of various shapes and localizes their contours accurately, reproducing the basic findings of object-based attention (Egly et al., 1994; Kimchi et al., 2007; see Mihalas et al., 2011). In this model, the G-cells encode only location, size and the rough shape of the object’s outline. Allowing only circular templates may be an oversimplification; the cortex might have a richer arsenal of templates, perhaps including elongated shapes.

The finding (Martin and von der Heydt, 2015) of increased spike synchrony between neurons whose border ownership preferences are consistent with the stimulating object, even when the neurons are widely separated in cortex, is strong evidence for feedback grouping circuits (**Figure 5**). The activation of grouping circuits might underlie the proto-objects of perception. There are two striking parallels: First, the intricate relationship between attentional modulation and border ownership selectivity (**Figure 3**; Qiu et al., 2007) indicating that these circuits provide a structure for object-based attention (Mihalas et al., 2011). Second, the persistence of border ownership signals (O’Herron and von der Heydt, 2009, 2011) parallels perceptual persistence (Landman et al., 2003). The finding that the persisting signals ‘remap’ (O’Herron and von der Heydt, 2013) shows that these grouping mechanisms enable the system to continue processing of an object across movements of the retinal image, which is the basis of object tracking (Pylyshyn and Storm, 1988) and ‘object-specific preview benefits’ in letter recognition tasks (Kahneman et al., 1992; Noles et al., 2005). Further experiments are needed to identify the postulated grouping circuits and future modeling studies to show if and how persistence and remapping can be implemented in the proposed neural grouping scheme.

## Conflict of Interest Statement

The author declares that the research was conducted in the absence of any commercial or financial relationships that could be construed as a potential conflict of interest.
